# Progressive Anterior Tibial Tumefaction: A Case of Unusual Bone Lesion

**DOI:** 10.7759/cureus.100248

**Published:** 2025-12-28

**Authors:** Catarina R Silva, João L Pereira, Nidia Oliveira, Elisa Veigas, Maria C Coelho

**Affiliations:** 1 Internal Medicine, Unidade Local de Saúde Viseu Dão Lafões, Viseu, PRT

**Keywords:** brown tumor, elective parathyroidectomy, hyperparathyroidism, osteitis fibrosa cystica, parathyroid gland adenoma

## Abstract

Brown tumors are rare, non-neoplastic bone lesions caused by excessive bone remodeling due to hyperparathyroidism. Their estimated prevalence is approximately 3-5% in primary hyperparathyroidism and about 1.5% in secondary forms. Prolonged elevation of parathyroid hormone (PTH) stimulates osteoclastic activity, leading to progressive bone resorption and cortical disruption. These lesions develop through replacement of normal bone marrow by highly vascularized fibrous tissue, often accompanied by repeated microfractures and hemorrhage, resulting in pain, skeletal deformity, structural weakness, and an increased risk of fractures. Their clinical and radiologic resemblance to malignancy often poses a diagnostic challenge.

We report the case of an 81-year-old male who presented with a progressively enlarging, painless tumefaction on the anterior aspect of the right tibia. Magnetic resonance imaging (MRI) of the lower limb revealed a large heterogeneous lesion with internal cystic areas, initially suggestive of a secondary bone process. Laboratory studies showed markedly elevated parathyroid hormone (PTH 705 pg/dL), hypercalcemia (11.5 mg/dL), increased alkaline phosphatase (ALP 416 U/L), and elevated prostate-specific antigen (PSA 11.88 ng/mL). Bone scintigraphy demonstrated diffuse uptake compatible with metabolic bone disease. Thoracoabdominal-pelvic computed tomography (TAP-CT) showed diffuse bone involvement, raising suspicion of metastatic disease, and a heterogeneous thyroid gland with small nodules in the right lobe. The possibility of primary thyroid neoplasia was ruled out after thyroid ultrasound and biopsy of the thyroid nodule were performed. Due to the elevated PSA, a PSMA-PET (prostate-specific membrane antigen positron emission tomography) was requested, which demonstrated diffuse bone involvement not compatible with metastasis from a primary prostate neoplasm. The free-to-total PSA ratio and digital rectal examination were consistent with benign prostatic hyperplasia. Due to elevated PTH and calcium levels, parathyroid scintigraphy was requested, which revealed a hyperfunctioning parathyroid nodule, compatible with a parathyroid adenoma. Biopsy of the tibial lesion confirmed a brown tumor, likely secondary to primary hyperparathyroidism caused by a parathyroid adenoma. The patient underwent a successful parathyroidectomy.

Although rare, brown tumors should be considered in the differential diagnosis of osteolytic bone lesions, especially in the presence of elevated PTH. Recognition of this entity is essential to prevent misdiagnosis and unnecessary oncologic treatments.

## Introduction

Brown tumor, despite its name, does not represent a neoplastic process but rather focal bony lesions resulting from excessive bone remodeling due to hyperparathyroidism [1-4]. It is a rare and pathognomonic manifestation of hyperparathyroidism that can occasionally be mistaken for malignancy [1,5]. The prevalence of brown tumors is estimated at approximately 3-5% in cases of primary hyperparathyroidism and around 1.5% in secondary hyperparathyroidism [6]. The condition occurs more frequently in women than in men and typically presents in the fifth to sixth decade of life [4].

Primary hyperparathyroidism is most often caused by a sporadic parathyroid adenoma, accounting for up to 85% of cases, while parathyroid carcinoma is a much rarer cause. In cases of carcinoma, it is essential to differentiate from skeletal metastases [4,5]. From a biochemical perspective, the main difference is that primary hyperparathyroidism causes an increase in serum calcium and reduced phosphate. Secondary hyperparathyroidism is characterized by hypocalcemia and hyperphosphatemia [4].

Magnetic resonance imaging (MRI) and bone biopsy assist in establishing the diagnosis. Ultrasound, 99mTc-sestamibi scintigraphy, and single photon emission computed tomography-computed tomography (SPECT-CT) can be used to identify parathyroid adenomas or neoplasms whenever primary hyperparathyroidism is suspected [4]. We present the clinical case of a brown tumor, a rare diagnosis that required extensive complementary evaluation of a bone mass, involving the exclusion of primary and secondary bone malignancies.

## Case presentation

**Figure 1:**
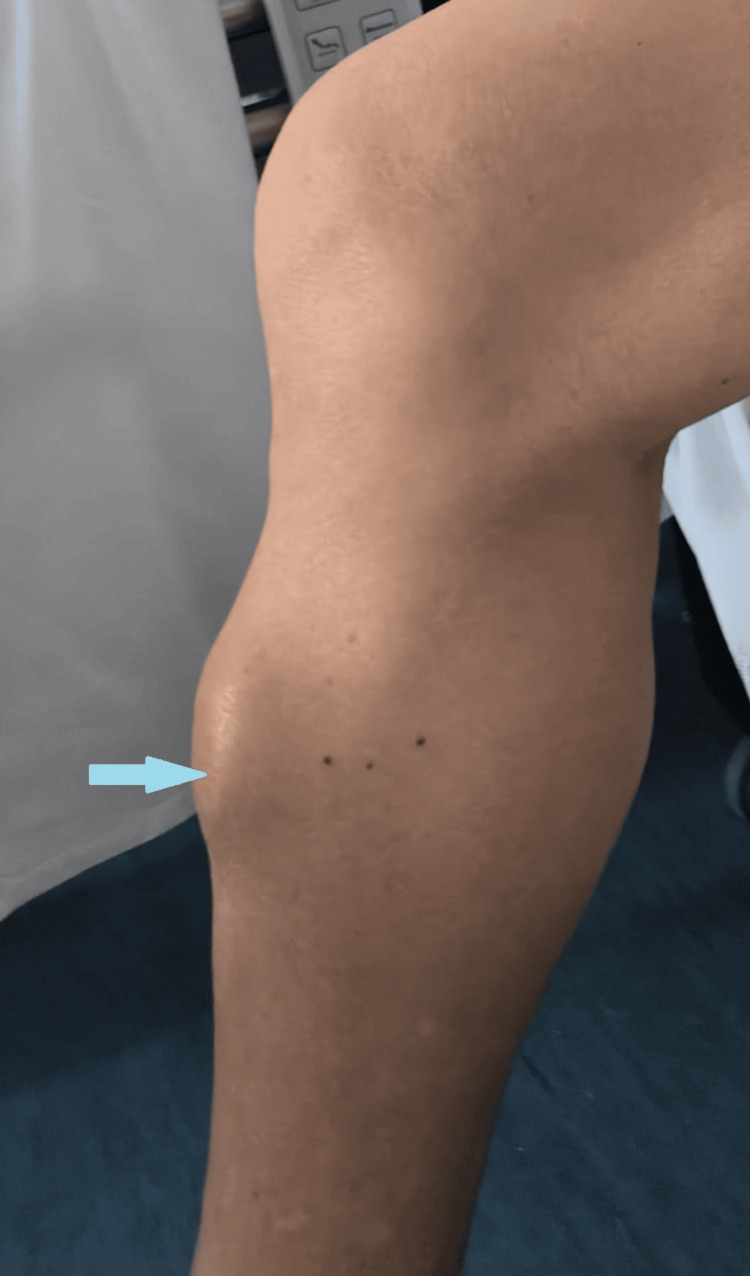
Image of the anterior tibial tumefaction.

**Figure 2:**
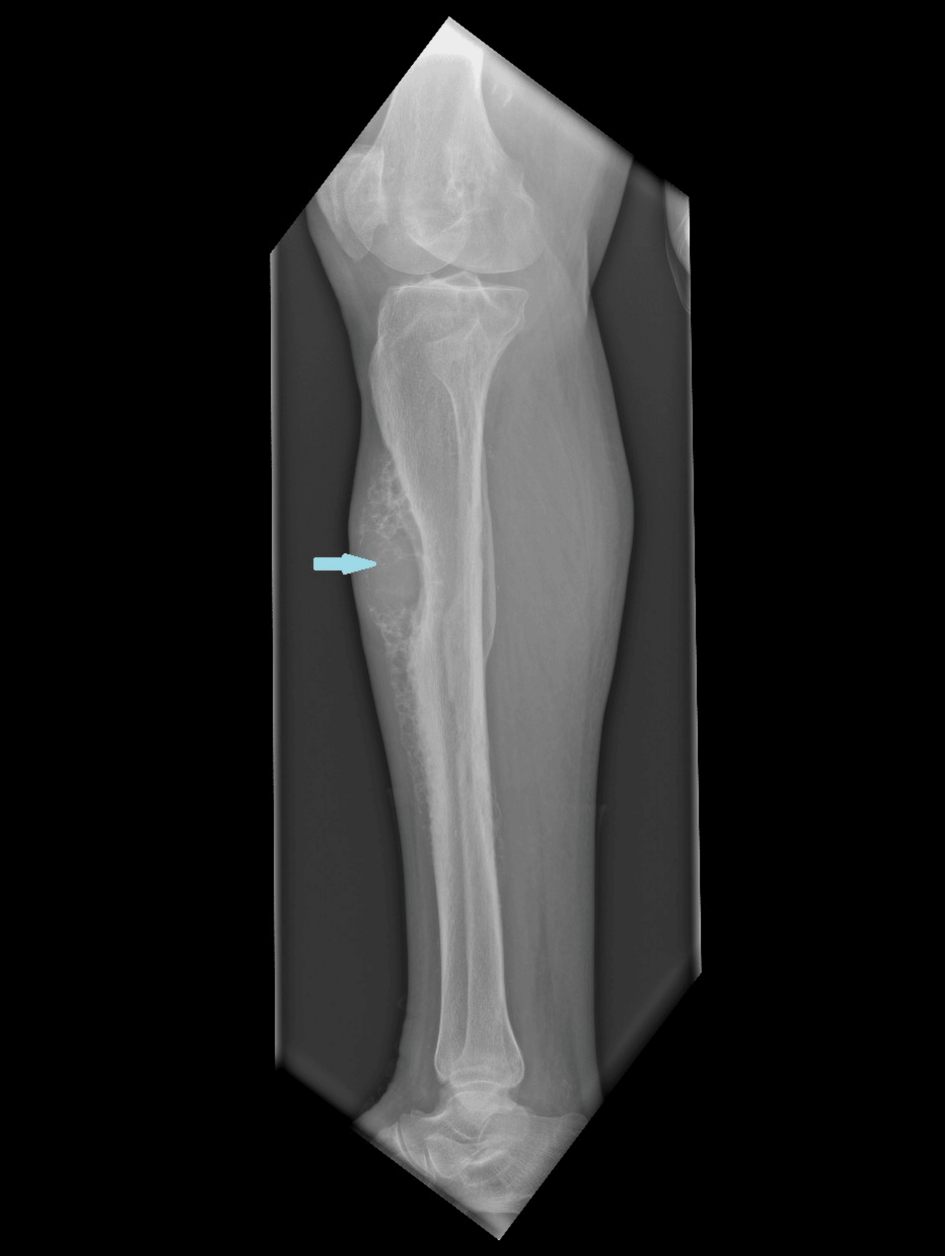
Radiograph of the lower limb showing a brown tumor as a tibial tumefaction with internal cystic areas.

An 81-year-old male patient with a past medical history of benign prostatic hyperplasia presented with a tumefaction on the anterior aspect of the right tibia, with a six-month history of progressive growth and rapid enlargement over the past two months. On physical examination, a hard, non-tender, non-inflammatory mass measuring approximately 7 cm in its largest diameter was noted on the upper third of the anterior tibial surface (Figures 1, 2).

Due to suspicion of a primary bone neoplasm versus bone metastasis, further investigations were carried out. Laboratory studies (Table 1) revealed normal thyroid function, markedly elevated parathyroid hormone (PTH 705 pg/dL), elevated serum calcium (11.5 mg/dL), elevated alkaline phosphatase (ALP 416 U/L), and elevated prostate-specific antigen (PSA 11.88 ng/mL).

**Table 1:** Etiologic analytical study of the tibial tumefaction ALP: alkaline phosphatase; GGT: gamma-glutamyl transferase; PSA: prostate-specific antigen; Free T4: free thyroxine; TSH: thyroid-stimulating hormone; PTH: parathyroid hormone

Etiologic analytical study			Etiologic analytical study		
Test	Results	Reference Values	Test	Results	Reference Values
Calcium/Phosphorus	11.5/2.4	8.7-10.4/2.3-3.7	Ratio Free/Total PSA	39%	
Albumin/Total protein	4.1/6.9 g/dL	3.5-5.0/6.6-8.7	Free T4/TSH	1.2/0.881 ng/mL	0.9-1.8
ALP/GGT	416/36 UI/L	25-100/7.0-49.0	Vitamin D	12.4 ng/mL	30-95
Total PSA/Free PSA	11.88/4.67 ng/mL	< 4.0	PTH	105.3 pg/mL	18.50-95.0
Normal protein electrophoresis					

**Figure 3:**
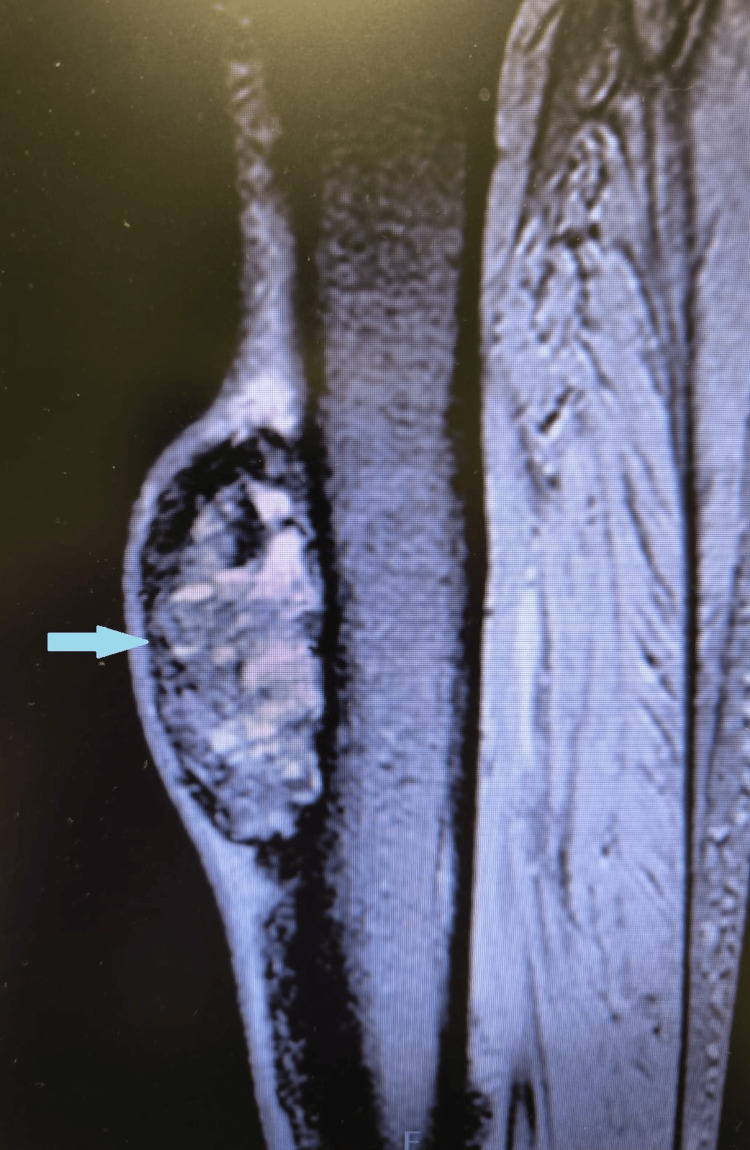
Sagittal MRI image of the lower limb demonstrating a tibial tumefaction measuring 13 cm in longitudinal length, with irregular contours, heterogeneous signal, and internal cystic areas.

Magnetic resonance imaging (MRI) of the lower limb demonstrated a large lesion along the anterior surface of the tibia, extending longitudinally for approximately 13 cm, with irregular contours, a heterogeneous signal, and internal cystic areas, suggesting a secondary lesion (Figure 3).

Bone scintigraphy showed diffuse bone uptake, more consistent with metabolic rather than neoplastic changes. To evaluate possible metastases from an unknown primary tumor, a thoracoabdominopelvic CT (TAP-CT) was performed, revealing a heterogeneous thyroid gland with small nodules in the right lobe and lytic lesions at the junction of the distal third of the right clavicle (21 mm), in the body of the sternum (23 mm), in the right acetabulum (29 mm), in the left iliac bone (12 mm), and in the D10 vertebra (15 mm). The overall findings suggested diffuse bone metastasis. Given the elevated PSA, a urology consultation was requested. The urologist reported that both the free-to-total PSA ratio and digital rectal examination were consistent with the patient’s known benign prostatic hyperplasia. A PET-PSMA (prostate-specific membrane antigen positron emission tomography) scan demonstrated diffuse hypermetabolic bone activity, suggesting widespread bone involvement, but the findings were not consistent with metastatic prostate cancer. Due to the thyroid abnormalities observed on TAP-CT, a thyroid ultrasound was performed, revealing a right-lobe TIRADS (thyroid imaging reporting and data system) 4 nodule measuring 19 × 15 mm, which was biopsied and found to be benign. Given the elevated serum calcium and PTH levels, a 99mTc-sestamibi scintigraphy was performed, revealing a hyperfunctioning nodule in the right parathyroid gland. A biopsy of the tibial lesion confirmed a brown tumor, likely in the context of primary hyperparathyroidism associated with a parathyroid adenoma. Based on the established diagnosis, the patient underwent parathyroidectomy.

## Discussion

Brown tumors represent a form of cystic osteitis fibrosa, considered the final stage of abnormal bone remodeling. They are benign, fibrotic, and erosive bone lesions linked to hyperparathyroidism, resulting from localized and accelerated osteoclastic activity that leads to replacement of bone by highly vascularized fibrous tissue, accompanied by repeated microfractures and microhemorrhages [1]. The name “brown tumor” originated from their gross histological appearance as a brownish mass composed of a combination of recurrent micro-fractures at various stages of remodeling with blood, hemosiderin, fibrous, and connective tissue [4]. These lesions usually develop in areas of pronounced bone resorption, most commonly in the metacarpals, phalanges, jaw, skull, pelvis, clavicle, ribs, femur, and spine [2,3,5,6]. Clinically, brown tumors may present with swelling, bone pain, or pathological fractures [1,2,4].

The radiographic appearance of brown tumors is variable; in some cases, the lesions are poorly defined, while in others, a sclerotic margin is visible. Multilobulated cystic changes are often present, and fractures are not uncommon. On MRI, brown tumors have been widely described as hypointense on T1-weighted images, with strong enhancement after administration of gadolinium-based intravenous contrast. On T2-weighted images, they may appear either hyperintense or hypointense. These imaging characteristics can also be seen in metastatic carcinoma, leukemia, and Langerhans cell histiocytosis, which can sometimes lead to misinterpretation of the lesions [3]. If there is any doubt in the diagnosis, a biopsy of the bone tumor is recommended for a definitive diagnosis [4].

Ultrasound is commonly used for patients with suspected parathyroid adenoma. The adenoma is frequently identified as a homogeneously hypoechoic lesion overlying the thyroid gland [4]. 99mTc-sestamibi scintigraphy can help confirm the presence and location of a parathyroid adenoma [4]. Hybrid imaging using single-photon emission computed tomography (SPECT) fused with the corresponding computed tomography (CT) is the preferred three-dimensional functional imaging technique for the localization of parathyroid tumors [4].

The preferred treatment is control of hyperparathyroidism. Tumor regression or even complete remission after parathyroidectomy has been well documented in both primary and secondary hyperparathyroidism [4,5]. The use of bisphosphonates can help manage hypercalcemia and reduce bone resorption, while the orthopedic approach focuses on stabilizing pathological fractures. Regular monitoring and follow-up are crucial to ensure effective treatment and recovery [6]. A review of the literature shows that there have been clinical cases in which patients underwent limb amputations due to suspected malignant bone tumors, with the final biopsy revealing a brown tumor. This highlights the importance of considering this entity to prevent unnecessary invasive or mutilating treatments for lesions that are ultimately benign [6].

The presented case highlights the diagnostic challenge of a bone mass. A lesion on the anterior tibia prompted evaluation for a primary bone neoplasm versus bone metastasis from an occult malignancy. Elevated total PSA raised concern for prostate cancer with bone metastasis, despite the patient’s known history of benign prostatic hyperplasia (BPH); however, this hypothesis was excluded based on a digital rectal exam, a free-to-total PSA ratio above 25%, and PSMA-PET, which showed bone lesions not consistent with prostate cancer metastasis. Thyroid nodules detected on TAP-CT and confirmed by thyroid ultrasound suggested possible thyroid cancer with bone metastases. This hypothesis was excluded by biopsy of the thyroid nodule, which was consistent with a benign lesion. Given the elevated serum PTH and calcium levels, compatible with primary hyperparathyroidism, parathyroid scintigraphy was performed and revealed a parathyroid adenoma. Bone biopsy showed a brown tumor, a benign lesion pathognomonic for hyperparathyroidism, confirming the link between the bone lesion and primary hyperparathyroidism. The patient underwent a successful parathyroidectomy and is under follow-up in general surgery.

## Conclusions

This clinical case highlights the diagnostic complexity of brown tumors. Documentation of this case underscores the importance of considering this rare entity in the differential diagnosis of osteolytic bone lesions, particularly in the context of elevated PTH and calcium levels. The discussion also included specific imaging studies and histological features that may aid in the prompt diagnosis of this clinical entity. Accurate recognition and appropriate management of these lesions are essential to prevent complications, such as pathological fractures, and to ensure that patients receive the correct treatment without unnecessary oncologic interventions.
